# Aggregated mesoporous nanoparticles for high surface area light scattering layer TiO_2_ photoanodes in Dye-sensitized Solar Cells

**DOI:** 10.1038/s41598-017-09911-w

**Published:** 2017-09-04

**Authors:** Kadhim Al-Attafi, Andrew Nattestad, Yusuke Yamauchi, Shi Xue Dou, Jung Ho Kim

**Affiliations:** 1 Institute for Superconducting and Electronic Materials (ISEM), Australian Institute for Innovative Materials (AIIM), University of Wollongong, North Wollongong, NSW 2500 Australia; 2 Department of Physics, College of Science, University of Karbala, Karbala, 56001 Iraq; 3 Intelligent Polymer Research Institute (IPRI), ARC Centre of Excellence for Electromaterials Science, AIIM, University of Wollongong, North Wollongong, NSW 2500 Australia; 4 World Premier International (WPI) Research Center for Materials Nanoarchitectonics (MANA), National Institute for Materials Science (NIMS), 1-1 Namiki, Tsukuba, Ibaraki, 305-0044 Japan

## Abstract

Hierarchically structured aggregates, consisting of TiO_2_ nanoparticles were produced via one-step solvothermal syntheses with a mixed solvent system containing both acetic acid and ethanol. Two of the resulting structures, one ~700 nm and the other ~300 nm in diameter, were found to be comprised of 8.5 nm and 10.5 nm anatase crystals, and possess specific surface areas of 138 and 106 m^2^ g^−1^ respectively. These particles were incorporated into Dye-sensitized Solar Cells (DSCs) as high surface area scattering layers, along with a layer of a transparent material. Solar-to-electric conversion efficiencies (PCE) of 9.1% and 8.2% were recorded using these aggregated particles as compared to those of commonly used large particles scattering layer 7.4%.

## Introduction

Since the breakthrough report by O’Regan and Gratzel in 1991, Dye-sensitized Solar Cells (DSCs) have attracted a great deal of research attention, due to their anticipated low-cost, simple manufacturing processes and promising photocurrent conversion efficiency^1–4^. A DSC consists of a number of components. Firstly, light is absorbed by a sensitizer to generate an excited state dye, which is capable of injecting electrons into the conduction band of wide band gap metal oxide, with these electrons being then transported through the metal oxide to an external circuit. After charge injection, the cationic sensitizer is reduced back to its neutral form by electrons donated from a redox mediator. Balance in this mediator is maintained by the catalytic counter electrode. The most commonly used materials in DSC for the above four components are organometallic ruthenium complexes, titanium oxide (TiO_2_), iodide/triiodide redox couple (I_3_-/I-) and platinum nanoparticles respectively^5, 6^. To date, the highest efficiencies of DSC have been recorded using TiO_2_ anatase nanoparticle photoanodes^3, 4^ due to excellent optoelectronic properties^7–9^, albeit with different sensitizers and redox electrolyte as compared to the above-mentioned system.

Meta-analysis shows that over 40% of research towards enhancing DSC performance has looked at modifying or developing an efficient photoanode nanostructure^6, 10–12^. In these studies it has been established that materials for efficient photoanodes should have (1) a large surface area to facilitate high dye loading, leading to high light harvesting efficiency, (2) have a well-connected network of pores for electrolyte diffusion^13^, (3) facilitate electron transfer (4) have a minimum of defects (both surface and bulk), including those formed at grain boundaries, to limit charge recombination energy losses^14^. These considerations are however somewhat contradictory. For instance, while decreasing the size of TiO_2_ nanoparticles increases the surface area, the average pore size is also decreased, limiting diffusion as well as leading to increased numbers of grain boundaries based defects^15^.

Another strategy to enhance the light harvesting efficiency is the use of light scattering effects as increasing the average path length of light as it travels through the TiO_2_ film, improving the probability of it being captured by a dye molecule (particularly in the wavelength range where the dye extinction coefficient is the lowest). According to Mie theory, the size of the scattering particle will determine the wavelengths of light which will be scattered efficiently^16^.

This is typically exploited by employing a bi-layer photoanode structure consisting of a transparent (weakly scattering), underlayer comprised of small particles and a layer of larger (scattering) particles on the top^12^. Sub-micrometre sized TiO_2_ spheres have been prepared by sol-gel methods by controlling the hydrolysis reaction and crystallized by subsequent calcination. This procedure has successfully obtained spherical TiO_2_ structures. However, their low surface area limits their application in DSCs^17–19^. Recently there has been a trend towards the production of hierarchical TiO_2_ structures, with large dimensions (effective scattering) consisting of nanoparticles (high dye loading)^15, 20–22^. Such previous solvothermal approaches used to synthesize hierarchically aggregated TiO_2_ nanoparticles had long synthesis procedures to control the morphology and/or crystalline phase^15, 17, 21, 23–25^.

These recent studies motive us to synthesize hierarchical mesoporous structures, with different aggregate sizes (300 ± 65 nm and 700 ± 150 nm, TiO_2_−300 and TiO_2_−700 respectively) composed TiO_2_ nanoparticles (~10.5 nm and ~8.5 nm respectively) in a facile solvothermal approach. We report a new and facile one-step solvothermal approach using titanium isopropoxide (TTIP) as a precursor in a solvent mixture containing acetic acid (AA) and ethanol (EtOH). Subsequently, we investigate their performance in DSCs, which is enhanced as compared to the commonly used, commercially available, light scatting layer (WER2-O). This is explained in terms of high surface area and relatively high light scattering, along with efficient electrolyte penetration through the highly interconnected mesoporous structure.

## Results and Discussion

**Figure 1:**
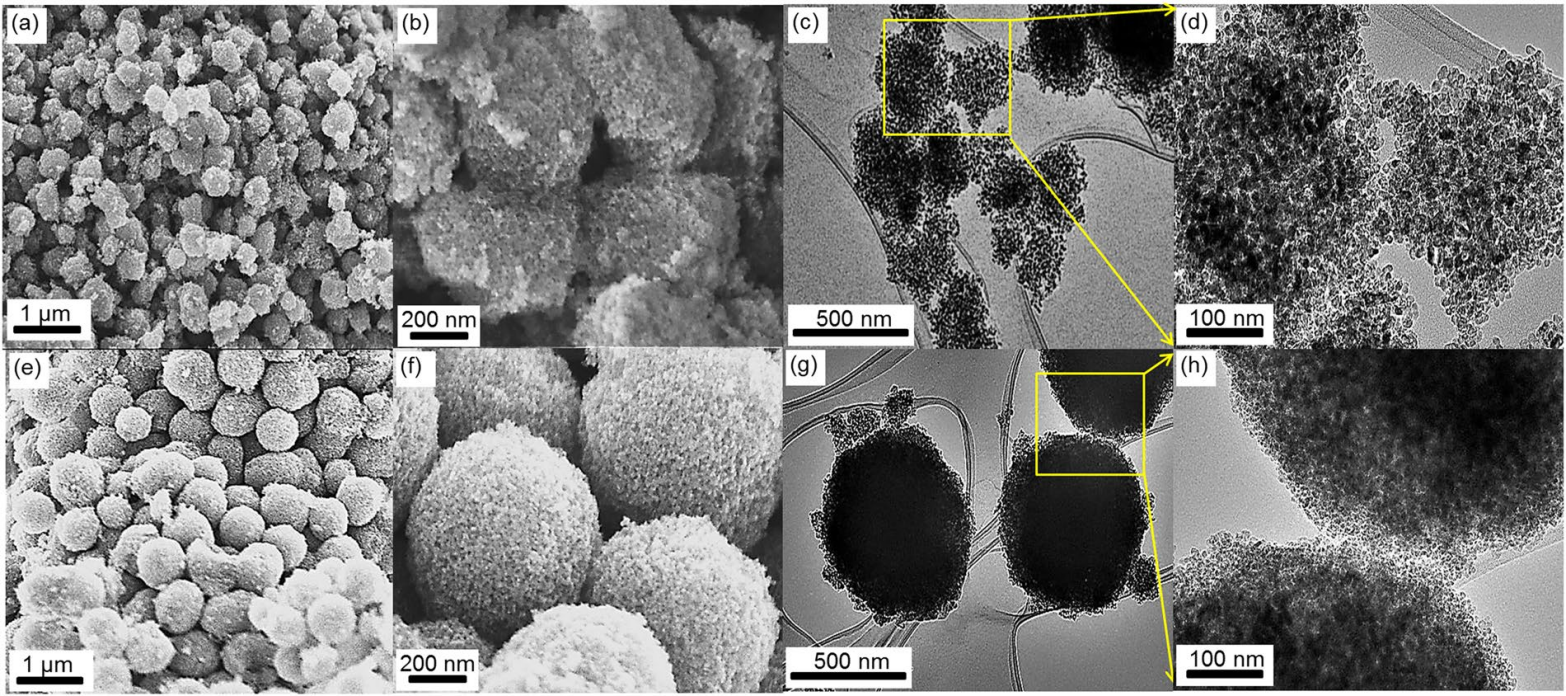
Structural (internal and morphological) characterizations of the calcined TiO_2_-300 and TiO_2_-700: (**a**–**d**) low and high magnification SEM and TEM images of TiO_2_-300; (**e**–**h**) low and high magnification SEM and TEM images of TiO_2_-700.

The morphologies and internal structures of the aggregated particles were characterized by scanning electron microscopy (SEM) and transmission electron microscopy (TEM). As shown in the high and low magnification (SEM) images (Fig. 1a,b,e and f). Hierarchical mesoporous structures, with different aggregate size (700 nm and 300 nm designated TiO_2_-700 and TiO_2_-300 respectively) composed TiO_2_ nanoparticles, were formed using mixtures of acetic acid and ethanol as a mixed solvent. SEM images also confirmed that (TiO_2_-700) and (TiO_2_-300) show highly connected mesoporous structure as a result of assembling TiO_2_ nanoparticles into hierarchical spheres and clusters shapes.

TEM images (Fig. 1c,d,g and h) show that both (TiO_2_-700) and (TiO_2_-300) have mesoporous structures, consisting of tightly interconnected and highly crystallized TiO_2_ nanoparticles with average sizes﻿ of (~8.5 nm) and (~10.5 nm) respectively.

**Figure 2:**
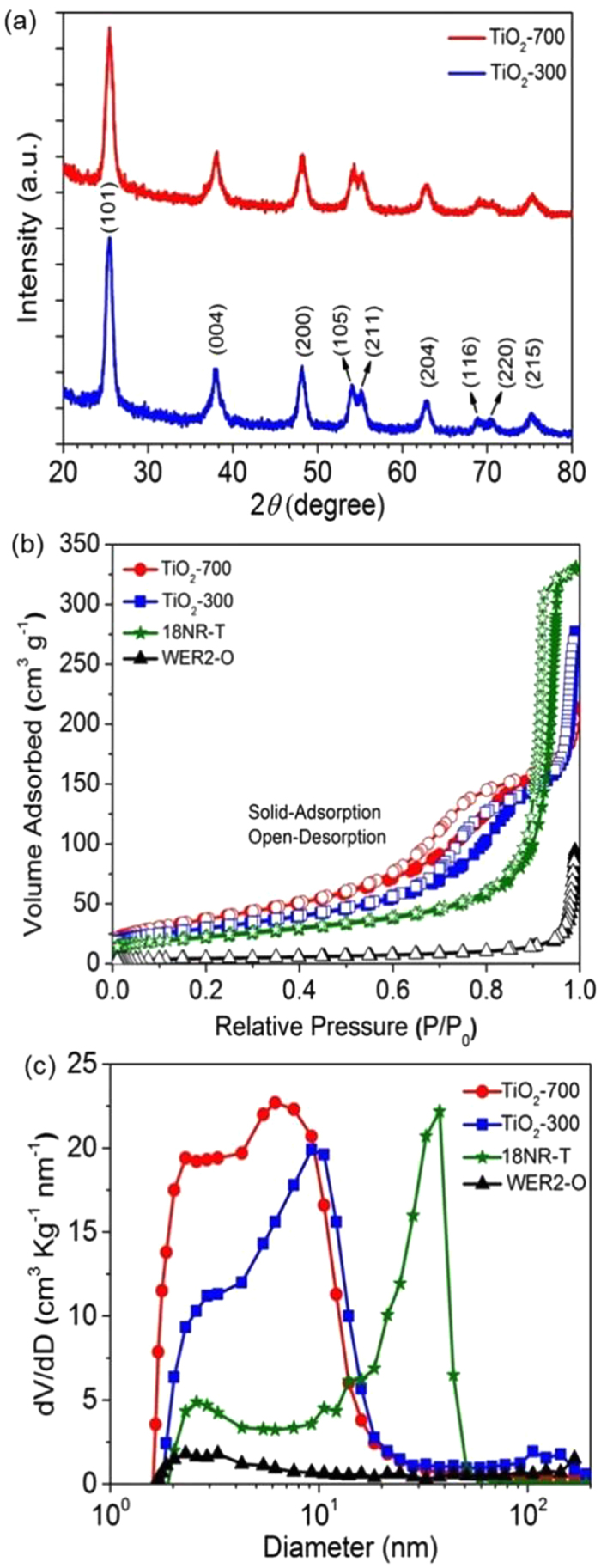
(**a**) X-ray diffraction patterns. (**b**) Nitrogen adsorption-desorption isotherms measurements. (**c**) Pore size distribution calculated from the adsorption branch of a nitrogen isotherm by the Barrett-Joyner-Halenda (BJH) method.

X-ray diffraction patterns (XRD) for the two aggregate materials are shown in (Fig. 2a). Both possess polycrystalline tetragonal anatase phase without any impurities or other phases (JCPDS no. 21–1272, a = 3.785 Å, b = 3.785 Å, and c = 9.514 Å) [Fig. S1 shows, this is even true before calcination]. The average crystallite sizes of TiO_2_-700 and TiO_2_-300 were ~8.5 nm and ~10.5 nm respectively, based on the Scherrer equation^26^. The high-resolution TEM (HRTEM) images [Fig. S2] confirmed that (TiO_2_-700) and (TiO_2_-300) are composed of nanocrystalline TiO_2_ with a fringe spacing of approximately (3.5 Å), corresponding to the (101) plane of the TiO_2_ anatase phase which is consistent with XRD analysis.

XRD, SEM and TEM analyses clearly demonstrated that both (TiO_2_-700) and (TiO_2_-300) have a hierarchical structure consisting of nano-sized TiO_2_ anatase nanoparticles, providing a highly interconnected mesoporous structure. This is verified by nitrogen adsorption/desorption measurements in (Fig. 2b and Table 1), which showed a type IV isotherm and H3 hysteresis loops at high relative pressures (P/P_0_ = 0.60–0.95). This indicates the presence of significant mesoporous structures in both (TiO_2_-700) and (TiO_2_-300) compared to (WER2-O) and is comparable with that of (18NR-T). Moreover, the hysteresis loops observed for the isotherms, even higher relative pressures (P/P_0_ = 0.85–0.95), indicate more condensed N_2_ in the pores and large voids of (TiO_2_-700) and (TiO_2_-300) compared to those of (WER2-O) and (18NR-T) leading to the conclusion that the overall surface area is larger for the aggregated particles. Barrett-Joyner-Halenda (BJH) analysis of pore size distribution (Fig. 2c) showed that the internal pore size (formed by aggregation nanoparticles) of (TiO_2_-700) and (TiO_2_-300) are (6.2 nm and 9.2 nm) respectively which are smaller than that of transparent layer (18NR-T) due to the smaller nano-size of their primary nanoparticles. However, the observed peak pore size of the Dyesol scattering layer (WER2-O) is around (2.7 nm). Due to the solid structure, this is assumed to arise from surface roughness. The external pore size related to the voids among (WER2-O) and (TiO_2_-300) particles are around 180 nm and 120 nm respectively due to owing approximately similar particle size. The external pore size of (TiO_2_-700) is expected to be around 350 nm, however, it is not observed here due to equipment limitations. Brunauer-Emmett-Teller (BET) calculations were conducted and summarized in Table 1. It is therefore expected that (TiO_2_-700) and (TiO_2_-300) would be capable of hosting a larger amount of dye, which can lead to higher photocurrent compared to those of (WER2-O). The internal and external pores of (TiO_2_-700) and (TiO_2_-300) can provide facile channels for the efficient electrolyte diffusion^15, 27^.

**Table 1:** Porosity (P), Specific surface area (S_A_) and Surface roughness factor (R_F_) of 18NR-T, WER2-O, TiO_2_-700 and TiO_2_-300 particles.

Sample	Porosity (%)^a^	Specific surface area (m^2^ g^−1^)	Roughness factor (µm^−1^)^b^
TiO_2_-300	63	106	154
TiO_2_-700	56	138	235
WER2-O	35	15	38
18NR-T	67	79	103

^a^The porosity calculated as: P = P_V_/(ρ^−1^ + P_V_), where P_V_ is the cumulative pore volume (cm^3^ g^−1^) and ρ^−1^ is the inverse of the density of anatase TiO_2_ (ρ^−1^ = 0.257 cm^3^ g^−1^). ^b^The estimated value of the surface roughness factor (R_F_) is calculated by R_F_ = ρ(1−P)S_A_^32^.

In addition, the aggregate size of around 700 nm and 300 nm, provide good scattering, while having high surface area true in undyed films, however, light travels in the film, hence increasing the probability of absorption light by the dye especially at wavelengths where the dye extinction coefficient is lower.

**Figure 3:**
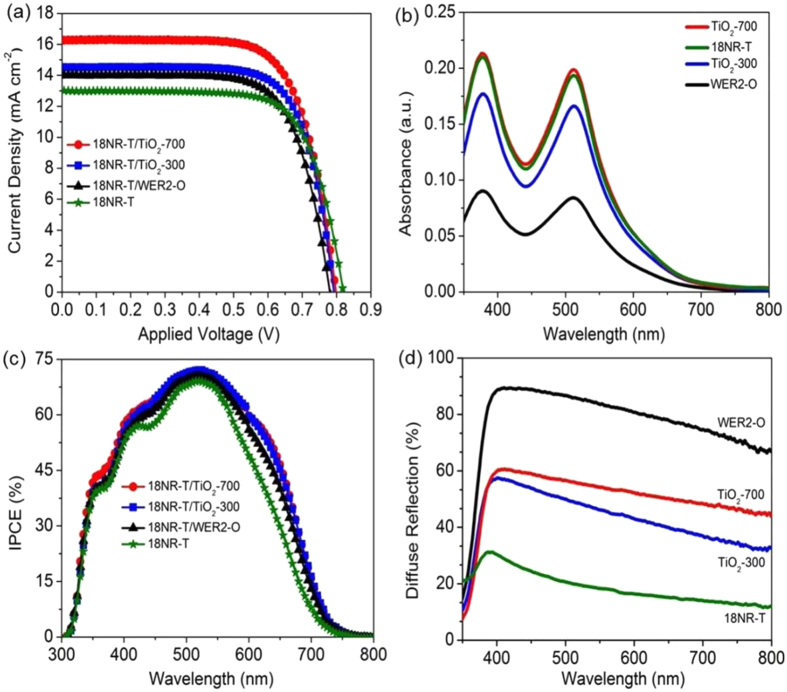
(**a**) J-V characteristics of DSC devices measured under 1 sun illumination with an area of 0.16 cm^2^; (**b**) absorbance spectra of the dye solution desorbed on the different scattering layers; (**c**) Incident photon to current conversion efficiency (IPCE) curves of DSCs﻿.

DSCs based on a bi-layer photoanode structure, incorporating a 18NR-T transparent layer, with either WER2-O, TiO_2_-700 or TiO_2_-300 as scattering layers, designated as (18NR-T/WER2-O), (18NR-T/TiO_2_-700) and (18NR-T/TiO_2_-300) respectively, along with a single layer (transparent only) (18NR-T) were prepared to investigate the effect of the scattering layers on the photovoltaic properties of the DSC. The current density-voltage characterisations (J-V) and key photovoltaic parameters are summarized in (Table 2) with representative J-V curves in Fig. 3a. DSC based on (18NR-T/TiO_2_-300) and (18NR-T/TiO_2_-700) photoanodes showed a significant enhancement in the photocurrent conversion efficiency compared to these using (18NR-T) or (18NR-T/WER2-O) photoanodes, with efficiencies of 8.2%, 9.1%, 7.2%, and 7.4% respectively. The higher efficiency of DSC devices based on (18NR-T/TiO_2_-300) and (18NR-T/TiO_2_-700) photoanodes is mainly due to enhanced *J*_*sc*_ while FF and *V*_*oc*_ are fairly consistent (Table 2). Dye loading on the (TiO_2_-700 and TiO_2_-300) films is significantly higher than that of (WER2-O) and comparable to that of Dyesol transparent layer (18NR-T) as seen from desorption experiments (Fig. 3b and Table 3). The hierarchical mesoporous structure of (TiO_2_-700) and (TiO_2_-300) based on high surface area aggregated nanoparticles can host more dye molecules, leading to higher *J*_*sc*_ while the very low surface area of (WER2-O) can result in lower *J*_*sc*_ due to the poor dye loading.

**Table 2:** J-V characterizations of DSC devices.

Device	*J*_*sc*_ (mA cm^−2^)	*V*_*oc*_ (V)	FF (%)	PCE (%)
18NR-T/TiO_2_-300	14.1 ± 0.4	0.79 ± 0.01	69 ± 1	8.2 ± 0.2
18NR-T/ TiO_2_-700	16.1 ± 0.1	0.80 ± 0.01	71 ± 1	9.1 ± 0.1
18NR-T/WER2-O	13.8 ± 0.3	0.79 ± 0.01	67 ± 1	7.4 ± 0.3
18NR-T	12.3 ± 0.7	0.83 ± 0.01	70 ± 1	7.2 ± 0.4

**Table 3:** The amount of dye on TiO_2_-300, TiO_2_-700, WER2-O and 18NR-T films.

Film	Dye loading (10^−7^ mol cm^−2^)	Dye loading (10^−5^ mol cm^−3^)
TiO_2_-300	0.55	14
TiO_2_-700	0.63	16
WER2-O	0.32	4
18NR-T	0.61	15

Electrochemical Impedance Spectroscopy (EIS) measurements were carried out to compare electron transfer and lifetime of devices based on bi-layer photoanodes. Nyquist plots of all the devices showed similar electrochemical interface impedance response. However, device based on (18NR-T/WER2-O) showed a more depressed arc in the second semicircle (lower frequencies) which is related to electron transfer at the TiO_2_ interface with FTO and the electrolyte (Fig. S3a and Table S1). Fittings for all these deives use CPE, as opposed to capacitive elements, in the model as the double layer interfaces between the electrolyte/photoanode are non-ideal and act as a leakage capacitor^28^.

Bode plots were used to estimated lifetime (τ = 1/2π*f*_max_)^29^ of injected electrons from dye through photoanode to the charge collector (FTO). (Fig. S3b) showed that the maximum value of frequency of devices based on (18NR-T/TiO_2_-700) and (18NR-T/TiO_2_-300) photoanodes were located at (20 Hz) and (25 Hz) respectively which is lower than that of (18NR-T/WER2-O) (38 Hz), implying that the lifetimes of electron transfer through (18NR-T/TiO_2_-700) and (18NR-T/TiO_2_-300) photoanodes are longer than in (18NR-T/WER2-O) due to reduced electron recombination and/or faster electron diffusion through high surface area hierarchical crystalline structure (there are more boundaries in the aggregates and increased surface area).

The enhancement in photocurrent densities and its relationship to enhanced light harvesting efficiency was also further investigated with an incident photon to current conversion efficiencies (IPCE) measurements. In Fig. 3c. devices based on (18NR-T/TiO_2_-300) and (18NR-T/TiO_2_-700) showed higher IPCE values in the entire measured wavelength range (300–800 nm) along with a broader shape around than those of (18NR-T) and (18NR-T/WER2-O), (Fig. 3c and Fig. S4), even though (WER2-O) itself was more scattering than the aggregates. Peak IPCE values were nearly identical, while more marked differences in the red part of the spectrum were seen, where the dye absorption is lower.

**Figure 4:**
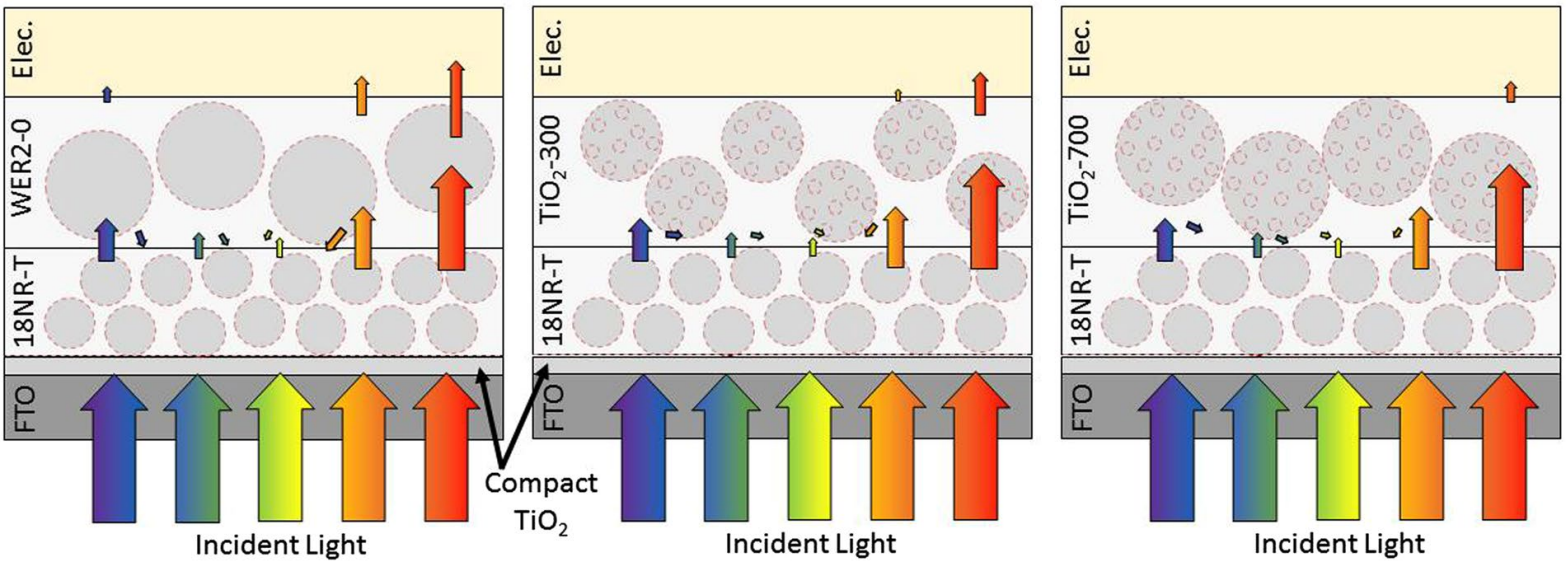
Schematic of DSC devices based on (18NR-T/WER2-O), (18NR-T/TiO_2_-700) and (18NR-T/TiO_2_-300) photoanodes with their multifunctional properties including dye loading, scattering light, and electrolyte diffusion trough mesoporous structure.

The light-scattering effect can be evaluated by measuring the diffuse reflection of photoanode films. Figure 3d shows the reflectance spectra of different photoanode films in the range of (400–800 nm). (18NR-T/WER2-O) photoanode showed the strongest diffuse reflection (65–85%) which is higher than that of (18NR-T/TiO_2_-700) and (18NR-T/TiO_2_-300) photoanodes respectively which are in turn, have higher diffuse reflection (40–60%) than that of transparent layer (18NR-T) (20–30%). The lower diffuse reflection of (18NR-T/TiO_2_-700) and (18NR-T/TiO_2_-300) (40–60%) compared to (18NR-T/WER2-O), is probably due to owning high porosity structure resulting in the less dense film (not being solid particles) (Table 1) and (Fig. 4).

## Conclusion

High PCE has been realized through the use of aggregated TiO_2_ structure as scattering layers. The sub-micro size hierarchical mesoporous spheres TiO_2_-700 comprised of 8.5 nm TiO_2_ nanoparticles, prepared by a simple one-step solvothermal method, provided the highest PCE of 9.1% in conjunction with a transparent TiO_2_ layer. This resulted from combined effects of higher dye loading, efficient electrolyte diffusion through the highly connected mesoporous structure and good light scattering properties. To the best of our knowledge, this is the highest efficiency for aggregated nanoparticles hierarchical microsphere as scattering layers with a commercial transparent TiO_2_ layer^22, 24, 30, 31^.

## Experimental

### Synthesis of TiO_2_-700 and TiO_2_-300

TiO_2_-700 and TiO_2_-300 were synthesized by a facile one-step solvothermal process. Briefly, Titanium isopropoxide (TTIP) (0.5 ml) was added dropwise to an acetic acid-ethanol mixed solvent under vigorous stirring for (1 h) at room temperature. A clear solution was formed which was transferred into a Teflon-lined stainless steel autoclave, 45 mL (Parr Instrument Company) heated to 180 °C (ramp time of 1 °C/min) for 9 h, after cooling down to room temperature the resulting white precipitate was collected and washed with distilled water and ethanol three times and then dried overnight at 90 °C. Finally, the samples were calcined at 400 °C (ramp time of 1 °C/min) in air for three hours. The morphologies, particle size and surface area of TiO_2_-700 and TiO_2_-300, were controlled by adjusting the acetic acid-ethanol volume ratio (AA:EtOH v/v) with keeping other solvothermal reaction conditions constant. The typical volume ratio of (AA: EtOH v/v) were used to synthesize TiO_2_-700, and TiO_2_-300 were (1:5 in ml) and (1:3 in ml) respectively.

### Preparation of photoanodes and DSCs assembly

Fluorine-doped tin oxide (FTO) glass was sequentially cleaned using soapy water, acetone, and ethanol in an ultrasonic bath for 20 min per solvent. A blocking layer of TiO_2_ was deposited on the cleaned FTO substrate using spray pyrolysis of titanium (IV) diisoproxide-bis-acetylacetonate (75 wt.% in isopropanol, Aldrich) solution (1:9 v/v in ethanol) at 450 °C. Scattering pastes (WER2-O, Dyesol Australia, TiO_2_-700 and TiO_2_-300 or as described below) were printed on the top of a single transparent layer (18NR-T, Dyesol Australia) using a Keywell screen printer with a custom mesh (43 T) to form 4 mm × 4mm (0.16 cm^2^) photoanodes. The printed transparent layer (18NR-T) was dried at 125 °C before scattering layers were deposited. Pastes of TiO_2_-700 and TiO_2_-300 were prepared using 1.0 g of TiO_2_ ground in a mixture of ethanol (25 ml), distilled water (1 ml), and acetic acid (0.2 ml). After that terpineol (5 g) and ethyl cellulose (0.5 g) were added to form a slurry which was sonicated and stirred for (2 h)^32, 33^. A viscous white paste was finally obtained after an evaporation process to remove water and ethanol. TiO_2_-700 and TiO_2_-300 scattering pastes were printed (thickness = 5.5 µm) on the top of a single transparent layer (18NR-T, Dyesol Australia) (thickness = 6.5 µm). For comparison, the photoanode including a single transparent layer (18NR-T) and a commercial scattering layer [Dyesol Australia WER2-O reflective Titania paste (thickness = 5.5 µm)] was printed. After that, the printed photoanodes were sintered using a multi-step program (up to 550 °C). Finally, the photoanodes were surface treated by soaking the photoanodes in (20 mM) aqueous solution of TiCl_4_ (Sigma) for 30 min at 70 °C, then washed and re-sintered at (500 °C for 30 min).

After cooling down to 110 °C, the photoanodes were immersed in an N719 dye solution (0.5 mM, Solaronix). The dye solution was a mixture of tert-butanol (LR, Ajax Chemicals) and acetonitrile (HPLC, Lab-scan) [1:1 v/v], the photoanodes were taken out from dye solution after 24 h and washed with acetonitrile and then dried. Counter electrodes were prepared by first drilling holes in a separate piece of FTO glass, to be used as a filling port for the electrolyte solution. One drop of (10 mM) H_2_PtCl_6_ solution (in ethanol) was smeared on the cleaned pre-FTO counter electrode and heating to 400 °C for 20 min. The counter electrodes are cooled before being sandwiched together with the photoanode, using a 25 µm Surlyn (Solaronix) spacer, by a hot press. The electrolyte solution [acetonitrile/Valeronitrile (85:15 vol %), iodine (I_2_) (0.03 M), 4-tertbutyl pyridine (4-tBP) (0.5 M), 1-butyl-3-methylimidazolium iodide (BMII) (0.6 M), and guanidinium thiocyanate (GuSCN) (0.1 M)] was introduced into the filling port by the vacuum back-filling technique, and the filling port was then closed with a piece of Surlyn laminated to aluminium foil.

### Material Characterizations

The crystalline structures of TiO_2_-700 and TiO_2_-300 were examined using X-ray diffractometer (Bruker Advance, 40 kV, 30 mA) (Cu Kα, λ = 1.5406 Å) in range (2θ = 20°–80° with scan rate (1°/min). The morphology and internal structure of samples were examined by field-emission scanning electron microscopy (FE-SEM) (JEOL JSM-7500) and transmittance electron microscopy TEM (JEOL JEM-2010). Brunauner-Emmet-Teller (BET) surface area, as well as BHJ porosity and pore volume values, were determined from data collected on (Microtrac Belsorp-mini) nitrogen adsorption-desorption equipment. The amount of dye on the different scattering layers was calculated by measuring the absorbance of dye desorbed from the films (thickness = 4 µm, area = 1 cm^2^) in (4 ml) of (0.1 M) NaOH solution (distilled water: ethanol 1:1 v/v) using a Shimadzu UV-3600 spectrophotometer. The light scattering properties (diffuse reflectances) were measured using an integrating sphere (ISR-3100) and the above spectrophotometer. A Veeco Dektak 150 Surface Profiler was used for the film thickness measurements. Photocurrent density-voltage (J-V) measurements were measured using a solar simulator with AM1.5 filter; set to 1 sun (100 mW/cm^2^, PV Measurements, Colorado). A QEX10 system from (PV Measurements) was used for the incident to photocurrent conversion efficiency (IPCE) measurements in 5 nm steps. The measured currents were referenced to a calibrated Si photodiode. A Reference 600 Potentiostat (GAMRY instrument) was used for electrochemical impedance spectroscopy measurements (EIS) which were carried out for DSCs based on different photoanodes under 1 sun illumination at *V*_*oc*_ in a frequency range (0.1–106 Hz) and AC voltage 10 mV.

## Electronic supplementary material

Supporting Information
