# Supplementary material for: Aggregated mesoporous nanoparticles for high surface area light scattering layer TiO2 photoanodes in Dye-sensitized Solar Cells
Source: Sci Rep. 2017 Sep 4;7:10341. doi: 10.1038/s41598-017-09911-w (PMC5583389; doi:10.1038/s41598-017-09911-w)
Supplement: Supplementary file 1 — Supporting Information [file 41598_2017_9911_MOESM1_ESM.pdf]

Supporting Information

Aggregated mesoporous nanoparticles for high surface area light scattering layer TiO_2 photoanodes in Dye-sensitized Solar Cells

Kadhim Al-Attafi, [1, 2] Andrew Nattestad*[3] Yusuke Yamauchi, [1, 4] Shi Xue Dou, [1] Jung Ho Kim *[1]

[1] Institute for Superconducting and Electronic Materials (ISEM), Australian Institute for Innovative Materials (AIIM), University of Wollongong, North Wollongong, NSW 2500, Australia.

[2] Department of Physics, College of Science, University of Karbala, Karbala 56001, Iraq.

[3]Intelligent Polymer Research Institute (IPRI), ARC Centre of Excellence for Electromaterials Science, AIIM, University of Wollongong, North Wollongong, NSW 2500, Australia.

[4]World Premier International (WPI) Research Center for Materials Nanoarchitectonics (MANA), National Institute for Materials Science (NIMS), 1-1 Namiki, Tsukuba, Ibaraki 305-0044, Japan.

* Corresponding authors: Jung Ho Kim and Andrew Nattestad

Email: jhk@uow.edu.au

Email: anattest@uow.edu.au

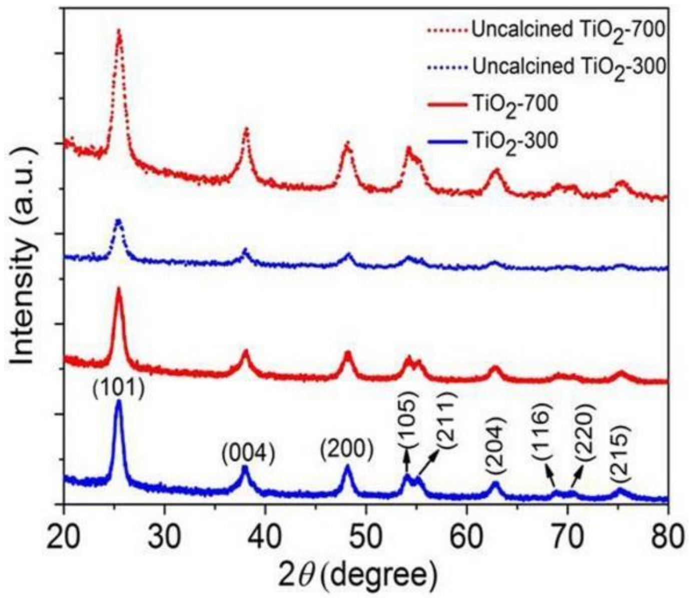

Figure 1: XRD diffraction patterns of TiO_2-700 and TiO_2-300 before and after calcination.

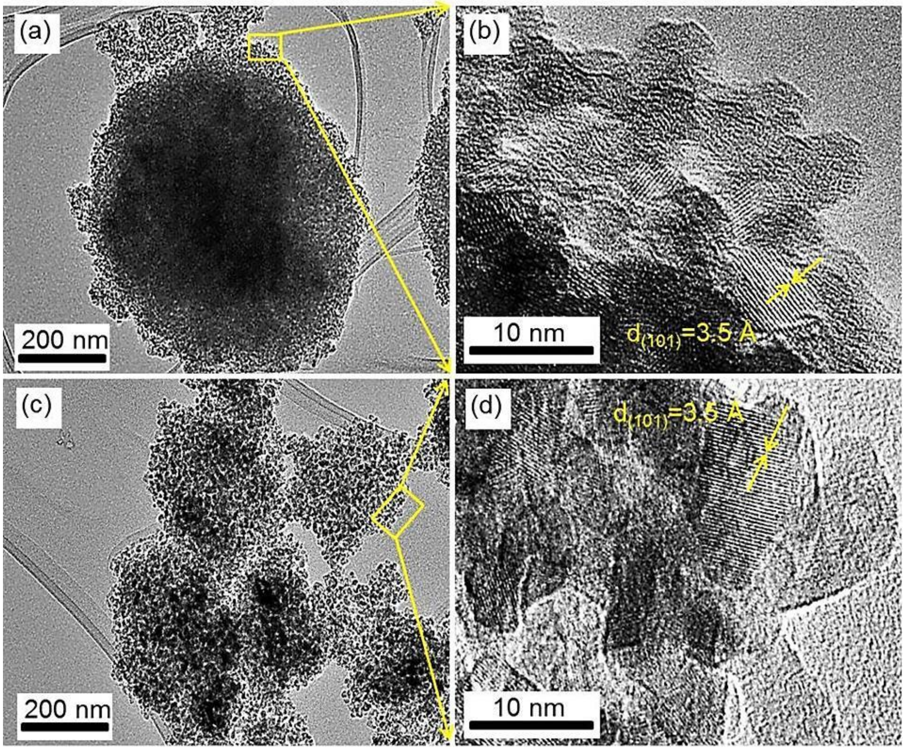

Figure 2: High magnification TEM and high resolution HTEM images: (a and b) of TiO_2-700; (c and d) of TiO_2-300.

Table 1: Electrochemical impedance spectroscopy data of DSCs based on 18NR-T/WER2-O, 18NR-T/TiO_2-700 and 18NR-T/TiO_2-300 photoanodes.

Devices	R_S (Ω)	R_{ct1} (Ω)	R_{ct2} (Ω)	CPE_2 (μF)
18NR-T/TiO_2-700	3.9	2.2	23.5	786
18NR-T/TiO_2-300	3.9	1.8	24.3	585
18NR-T/WER2-O	3.9	2.4	23.3	924

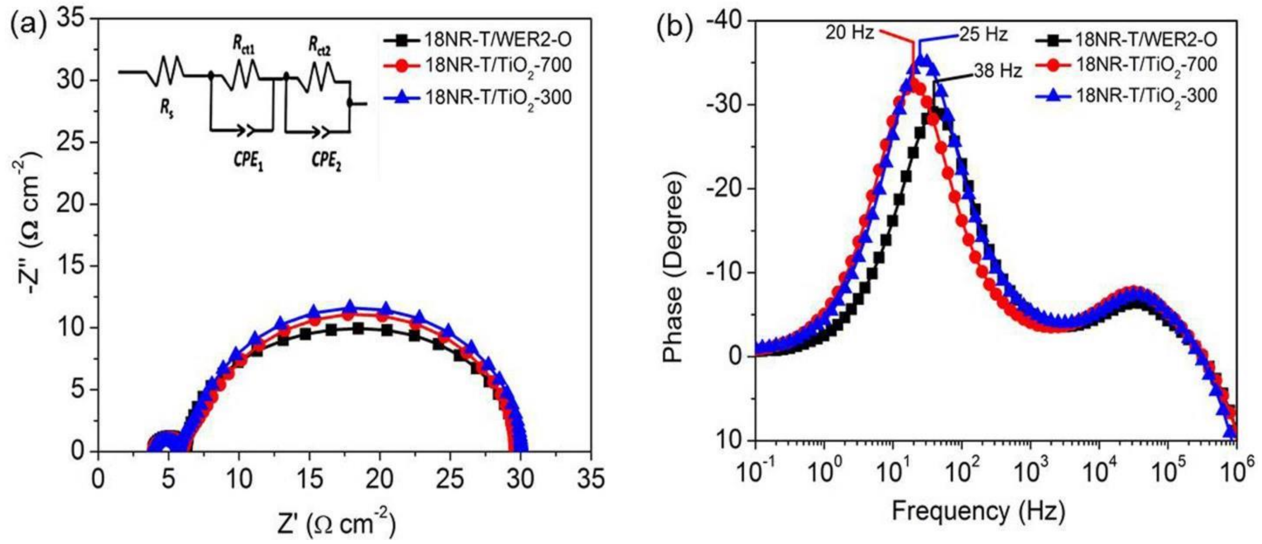

Figure 3: Electrochemical impedance spectroscopy; (a) Nyquist plot; (b) Bode plot of DSC based on 18NR-T/WER2-O, 18NR-T/TiO_2-700 and 18NR-T/TiO_2-300 photoanodes.

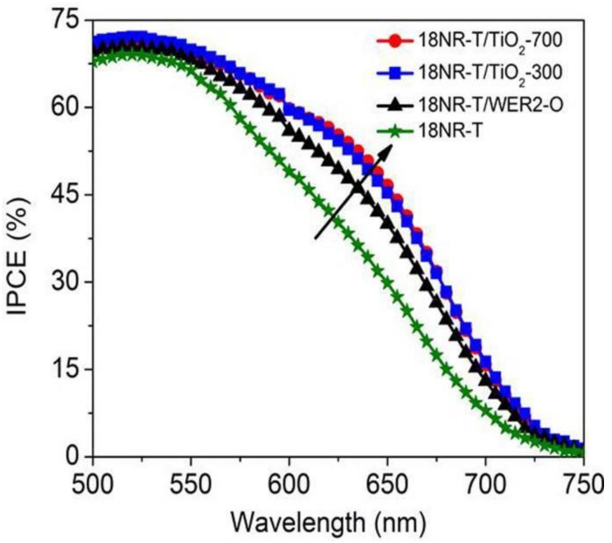

Figure 4: Broadening shape of (IPCE) spectra of DSC devices based on 18NR-T, 18NR-T/TiO_2-700, 18NR-T/TiO_2-300 and 18NR-T/WER2-O photoanodes.
